# Effect of some non-genetic factors on the productivity and profitability of Holstein Friesian dairy cows

**DOI:** 10.14202/vetworld.2021.242-249

**Published:** 2021-01-27

**Authors:** Amira M. Abd-El Hamed, Eman R. Kamel

**Affiliations:** Economics and Farm Management, Department of Animal Wealth Development, Faculty of Veterinary Medicine, Benha University, Moshtohor, Toukh 13736, Qalyubia, Egypt

**Keywords:** 305-milk yield, days in milk, days open, dry period length, Holstein Friesian, profitability

## Abstract

**Background and Aim:**

Milk yield (MY) is one of the main factors that affect the economic profitability of dairy farms. Thus, increasing the MY per animal and decreasing the feed cost can lead to economic gains, so the objective of this study was to evaluate the effect of dry period length (DPL), days open (DO), and days in milk (DIM) on the productivity and profitability of dairy cow farms.

**Materials and Methods:**

Data used in this study were taken from 3095 lactation records of Friesian dairy cows of private and governmental sectors. The data were classified into 4 DPL categories: DPL_1_ <45 days; DPL_2_ 45-60 days; DPL_3_ 61-75 days, and DPL_4_ >75 days, 3 DO categories: DO_1_ ≤90 days; DO_2_ 91-110 days and DO_3_ >111 days, and 8 DIM categories: DIM_1_ 180-210 days; DIM_2_ 211-240 days; DIM_3_ 241-270 days; DIM_4_ 271-300 days; DIM_5_ 301-330 days; DIM_6_ 331-360 days; DIM_7_ 361-447 days; and DIM_8_ >447 days.

**Results:**

The average net profit (NP) was significantly different (p<0.05) among different categories of DPL, DO, and DIM in both production sectors, where high estimates of NP were calculated for DPL_3_ (30667.3 EGP), and it was the lowest for DPL_1_ (19690.6 EGP). DO_2_ had the highest NP (30754.1 EGP), while DO_3_ had the lowest NP (24875.5 EGP). DIM_3_ had the highest NP (29569.3 EGP), while DIM_8_ had the lowest NP (19528.4 EGP).

**Conclusion:**

Finally, we can conclude that DPL 61-75 days, DO 91-110 days, and DIM 241-270 days had the highest level of total MY, total return, and NP. Private dairy cow farms achieve a higher level of NP than governmental ones under subtropical Egyptian conditions.

## Introduction

Cow is a dairy animal that plays a great role in milk production all over the world [1]. The performance of dairy cows in terms of fertility and milk production is the key to farm profitability [2]. Major environmental factors that may affect milk production and animal performance are production sector, calving season, and management [3]. Several studies showed that the dairy production sector affects milk yield (MY) significantly [4,5]. This might be due to variations in the level of management [6]. Milk production during a specified period of lactation is usually used as an indicator for dairy cow performance. A common measure is MY per lactation (total milk yield within 305 days [305-MY]) or daily milk yield (DMY). Furthermore, days in milk (DIM) can be of interest [7]. Lactation length is the period between two successive calvings during which cows are able to produce milk or lactate [8]. Lactation length is an important production trait as it affects the total MY. A lactation period of 305 days is commonly accepted as a standard [9]. Furthermore, the dry period length (DPL) plays an important role in preparations for calving, milk production, and dairy cow health. Dry period is called a resting period in which the udder is prepared for the next lactation. Enough management of this period optimizes the mammary gland and fetus development in the last month of pregnancy, as well as the milk production of the next lactation. This also has a positive effect on cows’ metabolic health and reproductive performance [10]. Number of days open (DO) was reduced, and first ovulation occurred earlier in cows with a shortened DPL [11]. Increasing the DPL leads to decreasing the total returns (TR) and increasing production costs and lowering the net profit (NP) [12]. However, optimal DPL may differ depending on herd size, parity number, and level of milk production among other factors [13,14]. Longer DPL decreases the annual production of animal by increasing it’s DO [15]. DO is the period between calving and successful mating [16]. It is used to assess the reproductive performance and to make an economical decision in the dairy herd [17]. It is a complex trait that is affected by many factors such as season of calving, parity, management policies, herd size, production level, and artificial insemination (AI) techniques [18]. Conception rate (CR) (S/C) directly determines the total herd profitability. Hence, to maximize the farm profitability, it is very important to increase the CR up to maximum level [19], reduce the cost of AI, and shorten the DO [20]. Improving the level of management is required for optimal reproduction performance [21].

To improve the productivity and profitability of dairy cows, it is necessary to study some variables that affect animal’s performance and farm economy. The aim of this study was to evaluate the effect of DPL, DO, and DIM on the productivity and profitability of governmental and private dairy cow farms under subtropical Egyptian conditions.

## Materials and Methods

### Ethical approval

The study was approved by the Committee of Animal Care and Welfare, Benha University, Faculty of Veterinary Medicine, Egypt (BUFVTM:03-07-20).

### Study period and location

The study was carried out through field surveys in different regions of dairy cow farms (Cairo and EL Sharkia governorates) during the period that extended from summer 2016 to winter 2019 on random samples of private and governmental production sectors.

### Animals and management

Data used in this study were estimated from 3095 lactation records of Holstein Friesian dairy cows. All animals on the farm were housed in free-stall shaded open yards, bedded with sand floor, and supplied with a cool spraying system during the summer season. Animals were grouped according to average DMY into fresh (from calving day till 60 days postpartum), high, medium, and low milk-producing cows. All groups of cows were fed a balanced total mixed ration, although the diet composition differed according to the region, sector, and management. Water was freely available at all times. Lactating cows were machine-milked 2 times per day, and milk production was recorded at each milking. The collected data were milk production records and reproduction records.

### Productive traits

They included 305-MY, DIM, DMY, DPL, and calves sales. 305-MY=305×Total MY/DIM [22,23]. DMY=Total MY per cow per lactation/DIM. DPL is defined as the number of days between the dry-off date and the subsequent parturition date [24,25]. Calves sales is the price of 1-day-old calf or calves added to a herd.

### Reproductive traits

They included S/C (number of insemination doses till conception) calculated individually for each cow and DO.

#### Economic indices

They included **c**alculations of costs and returns:

1. Fixed costs=Depreciation cost of building+Depreciation cost of animal+Depreciation cost of parlor [23].
2. Variable costs=Feed cost+Veterinary cost+Labor cost+Fuel cost [23].
3. Total costs (TC)=Fixed costs+Variable costs [26].
4. TRs=Returns from milk sales (Amount of kg milk produced×Price of kg milk)+Value of calves sold (Price of 1 day old calf)+Fecal matter (Amount of fecal matter produced m^3^×price of m^3^) [23].
5. NP=TRs–TC [27].

### Data classification

Data were classified into several categories to evaluate the productive, reproductive, and economic efficiencies of Holstein Friesian dairy cows. Data were classified according to DO, DIM, and days dry. DO was classified according to Arbel *et al*. [28] into DO_1_ ≤90 days; DO_2_ 91-110 days, and DO_3_ >111 days. DPL was classified according to El-Tarabany [29] into DPL_1_ <45 days; DPL_2_ 45-60 days; DPL_3_ 61-75 days, and DPL_4_ >75 days. DIM was classified according to Afzal *et al*. [30] into DIM_1_ 180-210 days; DIM_2_ 211-240 days; DIM_3_ 241-270 days; DIM_4_ 271-300 days; DIM_5_ 301-330 days DIM_6_ 331-360 days; DIM_7_ 361-447 days, and DIM_8_ >447 days.

### Statistical analysis

All statistical procedures were performed using the computer programs IBM SPSS/PC^+^ “version 23” [31]. Preliminary Levene’s test was performed to ensure the homogeneity of variances among groups. The general linear model procedure was used to analyze the productive, reproductive, and economic measures for each animal according to fixed continuous variables (DPL, DO, and DIM). Duncan’s multiple range test was used to test differences among means. Statistical significance between mean values was set at p≤0.05. Results are reported as means and standard errors. The model for statistical analysis comprised the fixed effects of DPL (4 levels), DO (3 levels), DIM (8 levels), and their interaction with the random effect of sector. The model included the following effects:


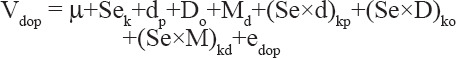


where:

V_dkop=_The response variable.

m=The overall mean of population.

Se_k_=Effect of k^th^ sector of production.

d_p_=Effect of p^th^ DPL.

D_o_=Effect of o^th^ DO.

M_d_=Effect of d^th^ DIM.

(Se×d)_kp_= Effect of the interaction between k^th^ sector and p^th^ dry period.

(Se×D)_ko_= Effect of the interaction between k^th^ sector and o^th^ DO.

(Se×M)_kd_= Effect of the interaction between k^th^ sector and d^th^ DIM.

e_dkop_=Un explained error term.

## Results

### Effect of DPL on subsequent MY and reproductive traits of Holstein Friesian dairy cows

Data summarizing results for the effect of DPL on subsequent DMY, 305-MY, DO, and S/C are included in Table-1. The DPL within both production sectors had a significant effect on DMY and 305-MY according to the four DPL categories. DMY and 305-MY values were (19.8-6046.9, 26.3-8036.7, 26.4-8060.3, and 23.1-7047.3 kg, respectively). The greatest yield was attained for private sector cows at DPL 61-75d, (DMY and 305-MY values were 34.2 and 10435.9 kg, respectively), while the lowest yield was for governmental sector cows at the shortest drying period length DPL <45 days, (DMY and 305-MY values were 10.7 and 3273.5 kg, respectively).

**Table-1:** Effect of DPL on subsequent milk yield and reproductive traits of Holstein Friesian dairy cows.

Trait	Sector	NO.	DMY	305-MY	S/C	Current DO
			
			Mean±SE	Mean±SE	Mean±SE	Mean±SE
DPL_1_	Private	58	28.9^b^±1.3	8820.3^b^±335.0	4.1^b^±0.4	176.9^b,c^±18.3
	Gov.	11	10.7^e^±3.0	3273.5^e^±769.3	3.1^b^±0.9	163.4^c^±42.0
	Total	69	19.8^C^±1.6	6046.9^C^±419.5	3.6^A,B^±0.5	170.1^C^±22.9
DPL_2_	Private	391	33.4^a^±0.5	10202.5^a^±129.0	3.8^b^±0.1	176.5^b,c^±7.0
	Gov.	131	19.2^c^±0.9	5870.8^c^±222.9	3.7^b^±0.2	219.6^a,b^±12.2
	Total	522	26.3^A^±0.5	8036.7^A^±128.8	3.7^AB^±0.1	198.1^B,C^±7.0
DPL_3_	Private	378	34.2^a^±0.5	10435.9^a^±131.2	4.0^b^±0.1	175.6^b,c^±7.2
	Gov.	292	18.6^c^±0.6	5684.8^c^±149.3	3.9^b^±0.2	246.5^a^±8.2
	Total	670	26.4^A^±0.4	8060.3^A^±99.4	4.0^B^±0.1	211.1^B^±5.4
DPL_4_	Private	181	31.5^a,b^±0.7	9619.2^a,b^±189.6	5.2^a^±0.2	228.0^a^±10.3
	Gov.	356	14.7^d^±0.5	4475.4^d^±135.2	4.2^b^±0.1	265.4^a^±7.4
	Total	537	23.1^B^±0.5	7047.3^B^±116.5	4.7^A^±0.1	246.7^A^±6.4

Means within the same column carrying different superscripts (small letters) are significantly different (p<0.05). Means within the same column carrying different superscripts (capital letters) are significantly different (p<0.05). (Gov.)=Governmental, NO=Number, DPL=Dry period length, DPL_1_ <45 days; DPL_2_ 45-60 days; DPL_3_ 61-75 days, and DPL_4_ >75 days, DMY=Daily milk yield, 305-MY=Total milk yield within 305 days, S/C=Service per conception, DO=Days open, SE=Standard error

DPL within both production sectors had a significant effect on S/C. Reproductive indices of cows were decreased as the DPL increased. According to the four DPL categories, S/C was 3.6, 3.7, 4.0, and 4.7, respectively. The highest value was for private sector cows at DPL >75 days (S/C value was 5.2), while the lowest value was for the governmental sector at DPL <45 days (S/C value was 3.1). Concerning the results of the effect of DPL on the current DO, the DPL within both production sectors had a significant effect on current DO. Regarding the four DPL categories, current DO values were 170.1, 198.1, 211.1, and 246.7 days, respectively. The longest DO value was for the governmental sector at DPL >75 days (265.4 days), while the shortest value was for governmental sector at DPL <45 days (163.4 days).

### Effect of DPL on economic indices

Data summarizing results for the effect of DPL on total variable cost (TVC), TC, TR, and NP are represented in Table-2. DPL within both production sectors had a significant effect on TVC and TC. According to the four DPL categories, TVC and TC were (26713.5, 29495.0-29626.4, 32579.9-29672.3, 32609.2 and 28533.8, 31540.1 EGP, respectively). The highest TVC value was for the private sector DPL 61-75 days (31566 EGP). The highest TC value was also for private sector (DPL 45-60 days, 34767.3 EGP), while the lowest TVC and TC values were estimated for governmental sector (DPL <45 days, 22634.6 and 25096.9 EGP, respectively). DPL within both production sectors had a significant effect on TR and NP. According to the four DPL categories, TR and NP were 49185.5, 19690.6-63110.8, 30531.0-63276.4, 30667.3 and 56181.7, 24641.6 EGP, respectively. DPL 61-75 days for private sector had the highest TR and NP values (80307.7-45541.4 EGP, respectively), while DPL <45 days for governmental sector had the lowest TR and NP values (29376.5-4279.5 EGP, respectively).

**Table-2:** Effect of DPL on economic indices of Holstein Friesian dairy cows.

Trait	Sector	No.	TVC	TC	TR	NP
			
			Mean±SE	Mean±SE	Mean±SE	Mean±SE
DPL_1_	Private	58	30792.4^a^±284.6	33893.1^a^±295.7	68994.6^b^±2344.9	35101.6^b^±1922.6
	Gov.	11	22634.6^d^±653.5	25096.9^d^±679	29376.5^e^±5384.6	4279.5^e^±950.8
	Total	69	26713.5^D^±356.4	29495.0^D^±370.3	49185.5^C^±2936.5	19690.6^C^±2738.7
DPL_2_	Private	391	31559.7^a^±109.6	34767.3^a^±113.9	78675.2^a^±903.1	43907.9^a^±658.9
	Gov.	131	27693.2^b^±189.4	30392.4^b^±196.8	47546.5^c^±1560.3	17154.1^c^±920.4
	Total	522	29626.4^B^±109.4	32579.9^B^±113.7	63110.8^A^±901.4	30531.0^A^±840.7
DPL_3_	Private	378	31566.0^a^±111.5	34766.3^a^±115.8	80307.7^a^±918.5	45541.4^a^±1980.4
	Gov.	292	27778.6^b^±126.8	30452.0^b^±131.8	46245.1^c^±1045.1	15793.1^c,d^±467.0
	Total	670	29672.3^A^±84.4	32609.2^A^±87.7	63276.4^A^±695.7	30667.3^A^±648.8
DPL_4_	Private	181	31391.2^a^±161.1	34673.0^a^±167.4	74586.9^ab^±1327.4	39913.9^a,b^±1037.6
	Gov.	356	25676.4^c^±114.9	28407.2^c^±119.4	37776.5^d^±946.5	9369.3^d,e^±424.5
	Total	537	28533.8^C^±98.9	31540.1^C^±102.8	56181.7^B^±815.2	24641.6^B^±760.2

Means within the same column carrying different superscripts (small letters) are significantly different (p<0.05), means within the same column carrying different superscripts (capital letters) are significantly different (p<0.05). (Gov.)=Governmental, NO=Number, DPL=Dry period length, DPL_1_ <45 days; DPL_2_ 45-60 days; DPL_3_ 61-75 days and DPL_4_ >75 days. TVC=Total variable cost, TC=Total cost, TR=Total return, NP=Net profit, SE=Standard error

### Effect of current DO length on MY and economic indices of Holstein Friesian dairy cows

Data summarizing results for the effect of the current DO length on DMY, 305-MY, TVC, TC, TR, and NP are presented in Table-3.

**Table-3:** Effect of current DO length on MY and economic indices of Holstein Friesian dairy cows.

Trait	Sector	NO.	DMY	305-MY	TVC	TC	TR	NP
					
			Mean±SE	Mean±SE	Mean±SE	Mean±SE	Mean±SE	Mean±SE
DO_1_	Private	451	33.8^a^±0.4	10302.0^a^±121.5	31392.0^a^±105.5	34534.3^a^±109.3	79372.5^a^±850.8	44838.1^a^±790.7
	Gov.	157	18.5^c^±0.7	5647.7^c^±206.0	27383.7^b^±178.8	30102.3^b^±185.3	45978.9^c^±1442	15876.7^c^±1340.1
	Total	608	26.1^B^±0.4	7974.9^B^±119.6	29387.9^A^±103.8	32318.3^B^±107.6	62675.7^B^±837.1	30357.4^B^±778.0
DO_2_	Private	165	34.2^a^±0.7	10417.9^a^±201.0	31279.0^a^±174.4	34453.1^a^±180.8	80178.0^a^±1406.6	45724.9^a^±1307.2
	Gov.	97	18.5^c^±0.9	5646.9^c^±262.1	27439.5^b^±227.4	30200.7^b^±235.8	45965.9^c^±1834.6	15765.3^c^±1704.9
	Total	262	26.3^A^±0.5	8032.4^A^±165.1	29359.3^B^±143.3	32326.9^A^±148.6	63072.0^A^±1155.9	30745.1^A^±1074.2
DO_3_	Private	1124	30.4^b^±0.2	9284.6^b^±8.0	31293.9^a^±66.8	34578.7^a^±69.3	72246.0^b^±538.9	37667.3^b^±500.8
	Gov.	1101	16.4^d^±0.2	5009.3^d^±77.8	26647.8^c^±67.5	29429.1^c^±70.0	41512.7^d^±544.5	12083.6^d^±506.0
	Total	2225	23.4^C^±0.2	7146.9^C^±54.7	28970.8^C^±47.5	32003.9^C^±49.2	56879.4^C^±383.1	24875.5^C^±356

Means within the same column carrying different superscripts (small letters) are significantly different (p<0.05). Means within the same column carrying different superscripts (capital letters) are significantly different (p<0.05. Gov.=Governmental, NO.=Number, DO=Days open, DO_1_ <91 days; DO_2_ 91-110 days and DO_3_ >110 days. DMY=Daily milk yield, 305-MY=Total milk yield within 305 days, TVC=Total variable cost, TC=Total cost, TR=Total return, NP=Net profit, SE=Standard error

DO within both production sectors had a significant effect on DMY and 305-MY. According to the three DO categories, DMY and 305-MY were 26.1, 7974.9-26.3, 8032.4 and 23.4, 7146.9 kg, respectively. The greatest yield was attained for private sector cows at DO: 91-110 days, (DMY and 305-MY values were 34.2 and 10417.9 kg, respectively), while the lowest yield was for the governmental sector at DO: >111 days (DMY and 305-MY values were 16.4 and 5009.3 kg, respectively).

Concerning the economic indices, DO within both production sectors had a significant effect on TVC and TC. According to the three DO categories, TVC and TC values were (29387.9, 32318.3-29359.3, 32326.9 and 28970.8, 32003.9 EGP, respectively). TVC value was the highest for private sector at DO: ≤90 days (31392.0 EGP), while the lowest value was for governmental sector at DO >111 days (26647.8 EGP). In responding to TC value, private sector had the highest value at DO 91-110 days (34453.1 EGP), while the lowest TC value was for governmental sector at DO >111 days (29429.1 EGP).

DO within both production sectors had a significant effect on TR and NP. According to the three DO categories, TR and NP were 62675.7, 30357.4-63072.0, 30745.1 and 56879.4, 24875.5 EGP, respectively. Private sector at DO 91-110 days had the highest TR and NP values (80178.0 and 45724.9 EGP, respectively), while governmental sector had the lowest values at the longest DO length DO >111 days (41512.7 and 12083.6 EGP, respectively).

### Effect of DIM within both production sectors on MY and economic indices of Holstein Friesian dairy cows

Data summarizing results for the effect of DIM on DMY, 305-MY, TC, TR, and NP are presented in Tables-4 and 5.

**Table-4:** Effect of lactation length (DIM) within both production sectors on milk yield of Holstein Friesian dairy cows.

Trait	Sector	NO.	DMY	305 MY
	
			Mean±SE	Mean±SE
DIM_1_	Private	26	32.4^a,b^±1.6	9884.9^a,b^±497.3
	Gov.	45	18.3^d^±1.2	5585.7^d^±378.0
	Total	71	25.4^B^±1.0	7735.3^B^±312.3
DIM_2_	Private	54	30.9^b^±1.1	9439.7^b^±345.1
	Gov.	128	19.1^d^±0.7	5830.6^d^±224.1
	Total	182	25.0^B,C^±0.7	7635.2^B,C^±205.7
DIM_3_	Private	157	33.2^a,b^±0.7	10143.0^a,b^±202.4
	Gov.	116	18.3^d^±0.8	5594.6^d^±235.4
	Total	273	25.8^A^±0.5	7868.8^A^±155.2
DIM_4_	Private	325	33.9^a^±0.5	10338.1^a^±140.7
	Gov.	138	17.3^d^±0.7	5294.5^d^±215.9
	Total	463	25.6^A,B^±0.4	7816.3^A,B^±128.8
DIM_5_	Private	276	33.8^a^±0.5	10318.2^a^±152.6
	Gov.	127	17.3^d^±0.7	5293.8^d^±225.0
	Total	403	25.6^A,B^±0.4	7806.0^A,B^±135.9
DIM_6_	Private	209	31.8^a,b^±0.6	9704.7^a,b^±175.4
	Gov.	129	17.6^d^±0.7	5376.6^d^±223.3
	Total	338	24.7^C^±0.5	7540.7^C^±142.0
DIM_7_	Private	371	31.6^a,b^±0.4	9652.1^a,b^±131.6
	Gov.	319	16.7^d,e^±0.5	5103.0^d,e^±142.0
	Total	690	24.2^D^±0.3	7377.5^D^±96.8
DIM_8_	Private	322	26.7^c^±0.5	8151.9^c^±141.3
	Gov.	353	14.6^e^±0.4	4472.1^e^±135.0
	Total	675	20.7^E^±0.3	6312.0^E^±97.7

Means within the same column carrying different superscripts (small letters) are significantly different (p<0.05). Means within the same column carrying different superscripts (capital letters) are significantly different (p<0.05). Gov.=Governmental, NO.=Number, DIM=Days in milk, DIM_1_ 180-210 days; DIM_2_ 211-240 days; DIM_3_ 241-270 days; DIM_4_ 271-300 days; DIM_5_ 301-330 days; DIM_6_ 331-360 days; DIM_7_ 361-447 days and DIM_8_ >447 days, DMY=Daily milk yield, 305-MY=Total milk yield within 305 days, SE=Standard error

**Table-5:** Effect of lactation length (DIM) within both production sectors on economic indices of Holstein Friesian dairy cows.

Trait	Sector	No.	TC	TR	NP
		
			Mean±SE	Mean±SE	Mean±SE
DIM_1_	Private	26	34226.5^a^±451.3.0	76431.0^a,b^±3481.1	42204.5^a,b^±3235.4
	Gov.	45	29898.5^b,c,d^±343.0	45555.7^d^±2646.0	15657.2^d^±2459.3
	Total	71	32062.5^B^±283.4	60993.4^B^±2186.3	28930.9^B^±2032.0
DIM_2_	Private	54	34227.2^a^±313.1	73328.0^b^±2415.5	39100.8^b^±2245.0
	Gov.	128	30468.2^b^±203.4	47258.0^d^±1568.9	16789.8^d^±1458.2
	Total	182	32347.7^A^±186.7	60293.0^B,C^±1440.1	27945.3^B,C^±1338.5
DIM_3_	Private	157	34491.0^a^±183.6	78257.6^a,b^±1416.6	43766.6^a,b^±1316.6
	Gov.	116	30219.6^b,c^±213.6	45591.6^d^±1648	15372.0^d^±1531.8
	Total	273	32355.3^A^±140.9	61924.6^A^±1086.6	29569.3^A^±1009.9
DIM_4_	Private	325	34597.4^a^±127.6	79623.4^a^±984.6	45026.0^a^±915.1
	Gov.	138	29511.5^d^±195.9	43513.0^d^±1511.0	14001.5^d^±1404.4
	Total	463	32054.4^B^±116.9	61568.2^AB^±901.7	29513.7^A,B^±838.1
DIM_5_	Private	276	34599.0^a^±138.5	79483.1^a^±1068.4	44884.1^a^±993.0
	Gov.	127	29437.9^d^±204.2	43506.2^d^±1575.1	14068.3^d^±1463.9
	Total	403	32018.5^B^±123.4	61494.7^A,B^±951.6	29476.2^A,B^±884.5
DIM_6_	Private	209	34598.9^a^±159.2	75182.8.0^a,b^±1227.8	40583.9^a,b^±1141.2
	Gov.	129	29840.2^b,c,d^±202.6	44083.6^d^±1562.8	14243.4^d^±1452.5
	Total	338	32219.5^A,B^±128.8	59633.2^C^±993.7	27413.6^C^±923.6
DIM_7_	Private	371	34851.6^a^±119.5	74816.6^a,b^±921.5	39965.0^b^±856.5
	Gov.	319	29711.6^c,d^±128.8	42166.7^d,e^±993.8	12455.1^d,e^±923.7
	Total	690	32281.6^A,B^±87.8	58491.7^D^±677.7	26210.0^D^±629.8
DIM_8_	Private	322	34218.5^a^±128.2	64324.4^c^±989.2	30105.8^c^±919.4
	Gov.	353	28803.2^e^±122.5	37754.0^e^±944.7	8950.8^e^±878.1
	Total	675	31510.8^C^±88.7	51039.2^E^±683.9	19528.4^E^±635.7

Means within the same column carrying different superscripts (small letters) are significantly different (p<0.05). Means within the same column carrying different superscripts (capital letters) are significantly different (p<0.05). Gov.=Governmental, NO.=Number, DIM=Days in milk, DIM_1_ 180-210 days; DIM_2_ 211-240 days; DIM_3_ 241-270 days; DIM_4_ 271-300 days; DIM_5_ 301-330 days; DIM_6_ 331-360 days; DIM_7_ 361-447 days and DIM_8_ >447days, TC=Total cost, TR=Total return, NP=Net profit

DMY and 305-MY values were significantly different among different categories of DIM within both production sectors. According to the eight categories of DIM, DMY and 305-MY values were (25.4, 7735.3-25.0, 7635.2-25.8, 7868.8-25.6, 7816.3-25.6, 7806.0-24.7, 7540.7-24.2, 7377.5 and 20.7, 6312.0 kg, respectively). The highest DMY and 305-MY values were attained for private sector at DIM 271-300 days (33.9-10338.1 kg) and DIM 301-330 days (33.8-10318.2 kg, respectively), while the lowest yield was for the governmental sector at DIM >447 days (14.6-4472.1 kg, respectively).

Regarding the economic indices, TC, TR, and NP values were significantly different among different categories of DIM within both production sectors. According to the eight categories of DIM, the TC, TR, and NP values were 32062.5, 60993.4 and 28930.9-32347.7, 60293.0 and 27945.3-32355.3, 61924.6 and 29569.3-32054.4, 61568.2 and 29513.7-32018.5, 61494.7 and 29476.2-32219.5, 59633.2 and 27413.6-32281.6, 58491.7 and 26210.0-31510.8, 51039.2 and 19528.4 EGP, respectively.TC value was the highest for private sector at DIM 301-330d (34599.0 EGP), while the lowest value was for governmental sector at DIM >447 days (28803.2 EGP). In correspondence with TR and NP values, the highest values were found for the private sector at DIM 271-300 days (61568.2 and 29513.7 EGP, respectively), while the lowest values were for the governmental sector at DIM >447 days (37754.0 and 8950.8 EGP, respectively).

## Discussion

The aim of this study is to evaluate the effect of DPL, DO, and DIM on the productivity and profitability of dairy cow farms. Regarding the effect of DPL within production sectors on the subsequent MY and reproductive traits, there was a significant effect of DPL within different production sectors on DMY and 305-MY. The greatest yield was attained for the private sector cows at DPL 61-75 days_,_ while the lowest yield was for the governmental sector cows at the shortest drying period length DPL <45 days. These results might be because a short dry period does not provide enough mammary gland regeneration; also very long DPL extends the time with no milk income and increases the possibility of over-conditioned cows [32]. This result agreed with Van Knegsel *et al*. [10], who found that shortening the drying period reduces milk production. Furthermore, El-Tarabany [29], O’Hara *et al*. [33] showed that there was a reduction in the MY at either extremely short or long DPL. On the other hand, some studies concluded that postpartum MY is not affected by a shortened DPL [34]. A longer DPL reduced the MY in that lactation, thus allowing the diversion of dietary energy to tissue gain, which give higher MY in the next lactation [35], also Metin Kıyıcı *et al*. [36] recorded that longer DPL have a positive effect on 305-MY, although shorter DPL practices have a better outcome for pregnancy ratios of the first insemination. Regarding the significant effect of DPL within both production sectors on S/C, the reproductive indices of cows were decreased as the DPL increased; the highest value was for private sector cows at DPL >75 days, while the lowest value was for governmental sector at DPL <45 days. This result is in accordance with Grummer *et al*. [37], who explained that reducing DPL to 28 or zero days, decreases days to first ovulation, and increases first service CR. Furthermore, Metin Kıyıcı *et al*. [36] concluded that DPL had a significant effect on the S/C (p<0.01). The greatest pregnancy ratio (53.0%) in the first insemination was obtained from DPL group ≤40 days and the lowest pregnancy ratio (30.8%) was obtained from DPL group 61 to 70 days. DPL significantly affected current DO in private and governmental sectors. The longest DO value was for the governmental sector at DPL >75 days, while the shortest value was for the governmental sector at DPL <45 days. This result agreed with Watters *et al*. [38] and Chen *et al*. [39], who concluded that shortening the DP increases the reproductive efficiency by shortening the time to first ovulation, reducing numbers of ovular cows, shortening DO, and improving fertility. Abdeltawab *et al*. [40] recorded that cows with DPL <40 days had the best intervals reproductive indices compared with that recorded for both standard and long DP. Concerning the effect of DPL on the economic indices, DPL within both production sectors had a significant effect on TVC and TC. The highest TVC value was for the private sector at DPL 61-75 days. Furthermore, the highest TC value was for private sector at DPL 45-60 days, while the lowest TVC and TC values were estimated for governmental sector at DPL <45 days_._ These results might be due to short DPL had a lower milk production in the next lactation due to less developed mammary glands, as the early involution of the mammary gland might be inhibited when the cow is dried off later in lactation and closer to parturition [41], consequently the feed cost will decrease, which represents the highest level of TVC. This result agreed with Sosa *et al*. [12], who recorded that short DP had a lower TVC than long DPL. DPL significantly affected TR and NP in private and governmental sectors. DPL 61-75 days for the private sector had the highest TR and NP values, while DPL <45 days for the governmental sector had the lowest TR and NP values. These results were attributed to the high level of milk production for cows within DPL 61-75 days. This result in accordance with Sosa *et al*. [12], who concluded that DPL 60-91 days had the highest TR and NP. In responding to the production sector, the private sector had a higher level of NP than governmental one; this might be due to managemental variation. Regarding the effect of DO within both production sectors on the MY, and reproductive traits. Current DO have a significant effect on DMY and 305-MY, the greatest yield was attained for private sector cows at DO 91-110 days, while the lowest yield was for the governmental sector at DO 91-110 days. These results agreed with Yamazaki *et al*. [42], who concluded that the total MY of a lactation was affected by DO. [20] Found that, when CR increased, number of AIs, DO, and DIM decreased, while annual MY increased. On the contrary Němečková *et al*. [43] recorded that the calving interval (CI) higher than 440 days (DO higher than 110 days) had the highest level of milk production than short CI. DO affected economic indices in private and governmental sectors. Current DO had a significant effect on TVC and TC. TVC value was the highest for the private sector at DO ≤90 days, while the lowest value was for the governmental sector at DO >111 days; on the other hand, TC for the private sector had the highest value at DO 91-110 days, while the lowest TC value was for the governmental sector at DO >111 days. These results in the same line with Meadows *et al*. [44], who explained that TVC and TC increased due to increasing the expenditures that were spent on infertility treatments, costs of feed consumption, and costs of decreasing DMY per extended DO especially after 110 days after calving. In responding to a dairy farm, the highest cost is the animal feed cost [45]. In responding to the significant effect of current DO within both production sectors on TR and NP, private sector at DO 91-110 days had the highest TR and NP, while governmental sector had the lowest values at the longest DO length DO >111 days. These results might be due to increasing costs of infertility problems treatment, costs of feed consumption, and costs of decreasing DMY per extended DO especially after 110 days after calving, at which the break-even point occurred. This result in accordance with Ahme [23], who concluded that the average profit of Holstein Friesian cow increased gradually till reach to maximum level around 110 days after calving then decreased again. Furthermore, Mengistu and Wondimagegn [21], Lehmann *et al*. [46], and Kidane *et al*. [47] reported that DO is the part of the CI, and longer DO reduced the farm profitability. Zahed *et al*. [48] concluded that farm profitability increased by shortening CI, and prolonging this period for 1 day causes a financial loss for the dairy farm. This may be due to the increasing value in milk income. Milk production is a major source of income for the farm. TVC for long CI is higher (p<0.01) than that of medium and short CI. This difference is mainly due to the feed cost, as it is the main component of TVC and therefore long CI more expensive than other levels. This agreed with Kidane *et al*. [47], who explained that DO is a part of the CI, longer DO associated with decreased profitability. DIM affected DMY, 305-MY and economic indices in private and governmental sectors. DIM had a significant effect on DMY, 305-MY, TC, TR, and NP. The highest DMY and 305-MY values were attained for the private sector at DIM 271-300d and 301-330 days, while the lowest yield was for the governmental sector at DIM: >447 days. In correspondence with TC value, it was the highest for the private sector at DIM 301-330 days, while the lowest value was for the governmental sector at DIM_8_. Regarding TR and NP values, the highest values were found for the private sector at DIM 271-300 days, while the lowest values were for the governmental sector at DIM >447 days. This result disagreed with Facó *et al*. [49] and Hossen *et al*. [50] who found that MY increased by increasing the lactation length.

## Conclusion

The results of our study concluded that DPL 61-75 days, DO 91-110 days, and DIM 241-270 days had the highest level of total MY, TR, and NP. Private dairy cow farms achieve a higher level of NP than governmental ones under subtropical Egyptian conditions.

## Authors’ Contributions

AMA collected and manipulated the data, and then analyzed. ERK drafted and revised the manuscript. Both authors read and approved the final manuscript.
